# The Role of Positive *Klebsiella* Culture in Revision Hip and Knee Arthroplasty

**DOI:** 10.3390/pathogens15020164

**Published:** 2026-02-03

**Authors:** Vinzenz Bussek, Marion T. Tödtling, Jennyfer A. Mitterer, Veronika Achatz, Selma Tobudic, Jochen G. Hofstaetter

**Affiliations:** 1 2nd Department, Orthopaedic Hospital Speising, 1130 Vienna, Austria; vinzenz.bussek@oss.at (V.B.); marion.toedtling@oss.at (M.T.T.); 2 Michael Ogon Laboratory for Orthopaedic Research, Orthopaedic Hospital Speising, 1130 Vienna, Austria; jennyferangel.mitterer@oss.at (J.A.M.); veronika.achatz@oss.at (V.A.); 3 Center for Anatomy and Cell Biology, Medical University of Vienna, 1090 Vienna, Austria; 4 Department of Orthopaedic and Trauma, Medical University of Graz, 8036 Graz, Austria; 5 Department of Medicine I, Division of Infectious Diseases and Tropical Medicine, Medical University of Vienna, 1090 Vienna, Austria; selma.tobudic@meduniwien.ac.at

**Keywords:** periprosthetic joint infection, *Klebsiella*, revision arthroplasty, Gram-negative bacteria

## Abstract

Gram-negative (GN) periprosthetic joint infections (PJIs) are being increasingly reported. However, the role of *Klebsiella* species in PJIs remains unclear. Therefore, we aimed to analyze the prevalence, clinical presentation, microbial spectrum, antibiogram, treatment strategies and outcomes of *Klebsiella*-associated PJIs. A total of 1925 culture-positive total joint revision arthroplasties (rTJA) were retrospectively reviewed at a single center. Patient data were extracted from our institutional arthroplasty and PJI database. We identified 20 *Klebsiella*-positive PJIs (hip/knee, 11/9), representing 1.0% of all culture-positive rTJAs. The cases were predominantly polymicrobial (80%) and chronic (50%). Notably, *Klebsiella* spp. was rarely detected as an initial infectious event but was predominantly identified in the context of revision or re-revision procedures, frequently in patients with prior or persistent PJIs. *Klebsiella pneumoniae* was the most frequent species, with 44% showing multi-drug resistance. The antimicrobial susceptibility of *Klebsiella* isolates showed high resistance to cephalosporines and penicillin, in contrast little to no resistance to meropenem, gentamicin and levofloxacin. The most common initial surgical intervention was a two-stage revision (65%). Infection control (Tier 1) was observed in 11%, while further intervention was needed in 56% (Tier 3). All patients who had already died were classified as Tier 4 (33%). *Klebsiella* spp. was detected in 10.0% of GN rTJAs and was mainly associated with complex revision settings rather than primary infections. It is often associated with chronic polymicrobial infections and high antimicrobial resistance. The outcomes were generally poor, highlighting the need for pathogen-specific treatment strategies and improved diagnostics.

## 1. Introduction

Periprosthetic joint infection (PJI) is a severe complication following total joint arthroplasty (TJA) and is mainly caused by Gram-positive (GP) bacteria. The proportion of Gram-negative (GN) bacteria accounts for 11.5%–23% of PJI cases and has been increasing in recent years [1,2,3]. GN bacteria show high rate of antibiotic resistance and are often associated with delayed diagnosis. Despite the poor prognosis associated with Gram-negative PJIs, most research has concentrated on Gram-positive pathogens, leaving a gap in the understanding of GN-related PJIs [3,4,5,6,7]. Among the GN pathogens, *Klebsiella* spp. is one of the most frequently identified causes of GN PJIs, being 7%–15% of cases [2,8,9]. *Klebsiella* species are Gram-negative, non-motile encapsulated bacteria that typically colonize mucosal surfaces such as the oropharynx and gastrointestinal tract. Due to their ability to form biofilms, *Klebsiella pneumoniae* poses a significant challenge in the treatment of infections [10,11]. The virulence of *Klebsiella pneumoniae* is largely due to its polysaccharide capsule, which helps it evade the immune system. It also demonstrates significant antibiotic resistance, particularly against beta-lactam antibiotics, due to the production of beta-lactamases [10].

Although *Klebsiella* spp. has been repeatedly reported in the context of Gram-negative PJIs, the existing literature often does not clearly distinguish between primary infectious events and infections occurring in the setting of revision surgery prior or persistent periprosthetic infections.

Previous case reports describe *Klebsiella* spp. as an etiologic agent of challenging PJIs; however, a systematic analysis of *Klebsiella*-associated PJIs is currently lacking [11,12,13,14,15,16,17].

In particular, it remains unclear whether *Klebsiella* spp. primarily acts as an initial causative pathogen or rather emerges as a secondary organism in complex revision settings characterized by prior infection, repeated surgical interventions and extensive antimicrobial exposure.

Therefore, we aimed to investigate the incidence, clinical characteristics, microbial spectrum, antibiogram, surgical procedures and outcome in patients undergoing total hip or knee arthroplasty revisions (rTHA, rTKA) associated with *Klebsiella*.

## 2. Materials and Methods

After obtaining approval from the institutional ethics board, we investigated our arthroplasty registry and PJI database. Overall, we identified 1925 culture-positive knee and hip revision arthroplasties performed between January 2008 and March 2025. We included all revision TJA (rTJA) with a positive culture for *Klebsiella* spp. For the classification of periprosthetic joint infections (PJIs), we followed the guidelines established by the International Consensus Meeting (ICM) 2018 [18]. PJIs were categorized as acute if symptoms began within three months post-implantation and as chronic if they developed after this timeframe. Patient-specific risk factors were evaluated using the McPherson classification [19] and the Charlson Comorbidity Index (CCI) [20]. Due to its retrospective, non-comparative design, this study provides Level IV evidence. It was reported in accordance with the guidelines for reporting observational studies, the strengthening the reporting of observational studies in epidemiology (STROBE) statement [21].

### 2.1. Microbiological Analysis

For the microbiological investigation, we analyzed preoperative synovial fluid or swabs, as well as intraoperative periprosthetic tissue samples, swabs and sonication fluid. Explanted devices were immediately placed into sonication containers, where saline solution was added to fully immerse the implants. The samples were then sonicated and vortexed as described by Trampuz [22]. Tissue and sonication fluid (0.1 mL) were processed for bacterial and fungal identification using standard microbiological procedures [23]. Antimicrobial susceptibility testing was performed using the BD system (Becton Dickinson and Company, Franklin Lakes, NJ, USA) according to the manufacturer’s protocol, with the results interpreted based on the European Committee on Antimicrobial Susceptibility Testing (EUCAST) guidelines.

Initial antibiotic treatment was administered according to institutional protocols and later modified based on the antibiogram results.

### 2.2. Follow-Up and Clinical Outcome

The minimum follow-up time was one year. The rates of treatment success and failure were determined using the Tier classification system [24]. Cases classified as Tier 1 were considered successful, including those where a spacer was implanted followed by successful reimplantation and complete infection resolution. On the other hand, all cases from Tier 2 to 4 were categorized as failures, which included any relapses of infection, reoperation or fatal outcome.

### 2.3. Statistical Analysis

Continuous variables are reported as means with standard deviations. Categorical variables were compared using the Chi-square test, while continuous variables were analyzed through binary logistic regression. A *p*-value of less than 0.05 was considered statistically significant. All statistical analyses were conducted using IBM SPSS version 26 (SPSS Inc., Chicago, IL, USA).

## 3. Results

From a total of 201 GN-positive rTJAs, 20 (10.0%) were positive for *Klebsiella*. This bacterial pathogen was observed in both hip and knee replacements, with 11 out of 20 cases (55%) involving hip arthroplasties and 9 out of 20 cases (45%) involving knee arthroplasties. Six (30%) *Klebsiella* cultures were obtained during primary revision procedures, while fourteen (70%) were collected during subsequent revisions. Six (30%) *Klebsiella*-positive cultures were obtained preoperatively via aspiration. These six positive findings could not be detected intraoperatively again. The median interval between primary implantation and microbiological detection of *Klebsiella* species was 1179 days (range: 16–7531 days). Detailed information can be found in Table 1.

All patients were classified based on the ICM criteria. Four fulfilled at least one major criterion and seven received six or more minor criteria points. In total, eleven out of twenty patients (55%) were defined as infected, seven cases as possibly infected (35%) and two as not infected (10%) at the time of *Klebsiella* sample collection. Overall, 90% of the patients were categorized as clearly or likely infected according to the ICM criteria. All patients with a monomicrobial infection had a maximum ICM score of five points.

Acute PJIs were identified in eight cases and chronic PJIs in ten cases. One patient suffered from a hematogenous infection due to a chronic foot infection on the contralateral side. Two patients had an unexpected positive intraoperative culture (UPIC).

The median follow up was 62.0 months (IQR 75.3). Seven patients had died at the time of investigation. Two patients had no recent outpatient records; they were therefore classified as lost to follow-up. For two patients, the infection occurred recently, and therefore the minimum follow-up period of one year was not met. They were excluded from further outcome analysis.

In total, 7 of the 20 patients were male and the other 13 female. The average age at time of the infection was 66.9 (SD 17.9) years. The average body mass index (BMI) was 29.4 kg/m^2^ (SD 4.9).

### 3.1. Microbial Analysis

In total, 479 samples were analyzed, of which 221 (46%) were positive for both GN and GP microorganisms. The average number of samples taken per operation was 3.9 (Table 2). Microbial analysis revealed that four cases (20%) were monomicrobial, while a majority of sixteen cases (80%) were polymicrobial. In only two patients, the identical *Klebsiella* strain was repeatedly isolated upon subsequent re-revision. Across 20 patients, *Klebsiella*-positive cultures were obtained a total of 22 times.

*Klebsiella pneumoniae* (*K. pneumonaie*) (*n* = 18) could be found more often than *K. oxytoca* (*n* = 4). In the analysis of polymicrobial infections, a total of 49 organisms were identified. Among these, Gram-positive bacteria accounted for the majority, with 32 isolates followed by 13 g-negative bacteria and 4 fungal organisms. The most frequently detected GP species included *Staphylococcus epidermidis* (*n* = 8), *Enterococcus faecalis* (*n* = 4), *Cutibacterium acnes* (*n* = 4) and *Corynebacterium* spp. (*n* = 4). *Methicillin-resistant Staphylococcus aureus* (MRSA) was found in two of the three *Staph. aureus* isolates. GN organisms comprised primarily *Citrobacter koseri* (*n* = 3), *Proteus mirabilis* (*n* = 3) and *Pseudomonas aeruginosa* (*n* = 3), with *Escherichia coli* detected in two cases. Fungal isolates included *Candida albicans* (*n* = 3) and *Candida parapsilosis* (*n* = 1).

Eight (44%) of the eighteen *K. pneumoniae* bacteria showed antibiotic resistance pattern. All of them produced extended-spectrum beta-lactamases (ESBL). Four were additionally classified as 3 Multi-Resistant Gram-Negative (3 MRGN) and one as 4 MRGN. One *K. oxytoca* (25%) out of four was as well-categorized as 3 MRGN (Table 3).

**Figure 1:**
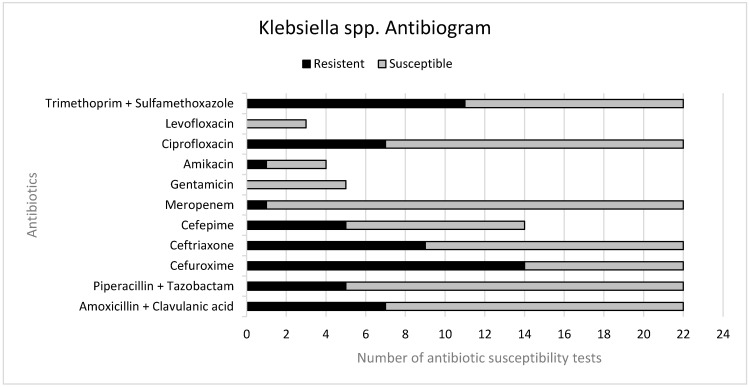
Resistance pattern of *Klebsiella* spp.

The antibiogram susceptibility testing revealed variable resistance patterns among *Klebsiella* isolates. Resistance to amoxicillin–clavulanic acid was observed in 7 of 22 isolates and to piperacillin–tazobactam in 5 of 22. Among cephalosporins, resistance was highest to cefuroxime (14/22), followed by ceftriaxone (9/22) and cefepime (5/22). Regarding fluoroquinolones, 7 of 22 isolates were resistant to ciprofloxacin, while none of the three tested showed resistance to levofloxacin. Trimethoprim–sulfamethoxazole non-susceptibility was observed in 11 of 22 isolates. Resistance to meropenem was uncommon (1/22), and none of the five tested isolates showed resistance to gentamicin (Figure 1).

At the time of *Klebsiella* sample collection, patients were receiving various antimicrobial agents. Cefuroxime was the most frequently administered agent (four cases). Fusidic acid, trimethoprim, rifampicin, daptomycin, dalbavancin, cefazolin and doxycycline were each administered twice. Ceftriaxone, piperacillin–tazobactam, teicoplanin, meropenem and linezolid were each administered once. Four patients did not receive any antimicrobial therapy at the time of *Klebsiella* sampling.

*Klebsiella*-directed antimicrobial therapy most frequently included meropenem (*n* = 8) followed by piperacillin–tazobactam (*n* = 4), ceftazidime/avibactam (*n* = 3), cephalosporins (*n* = 3) and fluoroquinolones (*n* = 3). In total, 8 out of 20 patients received sequential or multiple *Klebsiella*-directed antimicrobial regimens, whereas the remaining patients were treated with single-agent therapy or had incomplete treatment documentation.

In cases with a documented treatment duration (*n* = 15), the median duration of *Klebsiella*-directed antimicrobial therapy was 62 days (range, 7–226 days).

### 3.2. Surgical Procedures and Outcome

For the initial septic revision, three different therapeutic approaches were chosen: single-stage exchange, two-stage exchange or debridement with antibiotic therapy and implant retention (DAIR). In two cases (10%), an unexpected positive intraoperative culture (UPIC) was observed, and no further septic revisions were needed. The most chosen therapeutic approach was the two-stage exchange, which was performed in 13 cases (65%). In four patients (20%), a DAIR procedure was performed, and only one patient (5%) initially received a single-stage exchange.

**Figure 2:**
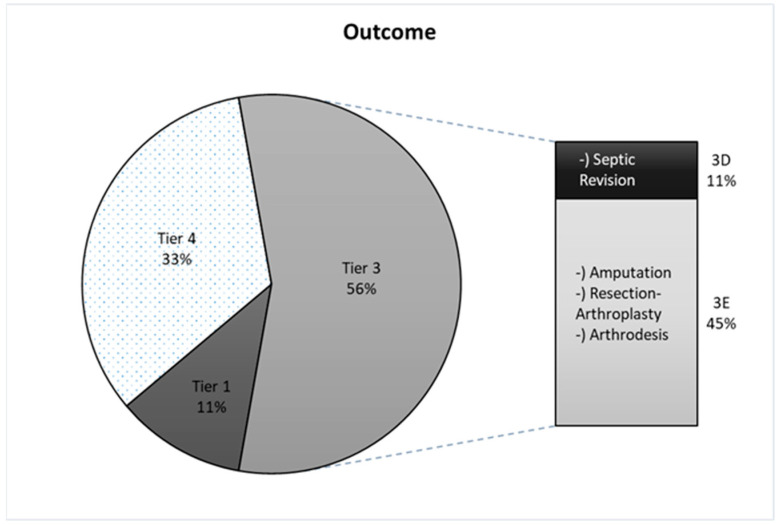
Outcome according to the TIER Classification. Tier 1: Infection control without continued antibiotic therapy. Tier 2: Infection control with the patient on suppressive antibiotic therapy. Tier 3: Need for reoperation or spacer retention. Tier 4: Death [23].

Based on the Tier classification, the outcome of the cases was assessed. Two patients (11%) were classified as Tier 1 and therefore were considered successfully treated. The majority of patients (10/18, 56%) were classified as Tier 3. Tier 3 includes extensive complications such as septic revision, amputation, resection arthroplasty and arthrodesis. Six patients were classified as Tier 4. In the first year, two died after primary revision (4a) and the other four died one or more years afterwards (4b) (Figure 2). Detailed information on individual case characteristics is provided in the Supplementary Material (Table S1).

## 4. Discussion

The findings from this study provide a detailed analysis of the role of *Klebsiella* species in PJIs following total hip and knee arthroplasties (TJAs). Although GP bacteria have been the focus of research on PJIs, the rising prevalence of GN bacteria warrants increased attention. In our cohort of 1925 revision arthroplasties, *Klebsiella* spp. was identified in 20 cases, representing 1.0%. Veghel et al. (1.7%) and Benito et al. (2.5%) reported slightly higher numbers for the presence of *Klebsiella* in PJIs [1,25]. In this revision database, 201 cases were associated with GN bacteria, with *Klebsiella* accounting for 10.0% of all GN cases. Other authors have reported similar values for *Klebsiella* in GN rTJAs ranging from 7% to 15% [2,8,9].

An important finding of this study is that *Klebsiella* spp. was rarely identified as a primary infectious event. Instead, most cases occurred in the context of revision or re-revision arthroplasties, frequently in patients with a history of prior or persistent periprosthetic joint infection. This suggests that *Klebsiella* may more often represent a secondary pathogen emerging in advanced stages of infection rather than an initial causative organism.

The predominance of *Klebsiella* spp. in late-stage and revision-associated PJIs may be explained by a combination of antibiotic-driven selection pressure and cumulative healthcare exposure. Repeated or prolonged antimicrobial regimens primarily targeting GP organisms may suppress susceptible flora and facilitate the emergence of GN pathogens such as *Klebsiella* spp. In addition, revision and re-revision procedures themselves represent independent risk factors for nosocomial GN acquisition due to repeated surgical interventions. Ramadanov et al. demonstrated in a systematic review that prolonged operative time and patient-related vulnerability may represent risk factors for bacterial infections [26]. Both factors are commonly encountered in revision surgery. The high proportion of polymicrobial infections observed in our cohort (80%) further supports the concept that *Klebsiella* spp. often acts as a secondary or complicating pathogen rather than a primary causative organism.

Polymicrobial infections occurred with similar frequency in hip and knee arthroplasties (*p* = 0.285). This finding is in line with the observation by Karczewski et al., as well Achatz et al. at our institution, who reported a high prevalence of polymicrobial patterns in Gram-negative PJIs [27,28]. Persistent infections caused by *Klebsiella* species were infrequent, with only two cases showing Klebsiella infection across two different microbiological investigations. This could be due to the dynamic nature of microbial populations in revision surgeries, where microorganisms often change and are frequently associated with polymicrobial infections, making it more challenging to detect specific pathogens and more likely for multiple revision surgeries [23,29,30]. Previous research has also indicated that shifts in the microbial spectrum observed during revision surgeries should not necessarily be regarded as new infections. Instead, these changes may reflect infections that were previously undetected in earlier stages of treatment [23].

In 6 out of 20 cases (30%), a positive *Klebsiella* culture was only detected in preoperative samples, which could not be confirmed intraoperatively. A possible explanation for this may be the initiation of antibiotic therapy after sample collection in the outpatient setting and before surgery, which can significantly reduce the likelihood of pathogen detection in subsequent cultures. In chronic complex cases, it may therefore be advisable to intentionally refrain from administering antibiotics during a defined interval before surgery in order to avoid compromising the diagnostic yield of intraoperative cultures.

Regarding the *Klebsiella* species identified, *K. pneumoniae* was the most prevalent, followed by *K. oxytoca*. In our study, we also found a high proportion of strains exhibiting antibiotic resistance. Extended-spectrum beta-lactamase (ESBL) production was observed in eight cases. Five strains were classified as 3 MRGN (multi-resistant Gram-negative) and one as 4 MRGN, which poses significant challenges for treatment due to their resistance to multiple antibiotic classes. The formation of biofilms from *Klebsiella* species provides an additional protective environment that enhances bacterial survival, promotes persistence and reduces susceptibility to antibiotics. When multiple organisms coexist within a biofilm, interspecies interactions can further increase resistance and tolerance, making eradication even more difficult [10,11]. None of the eight antibiotic resistant cases achieved a successful outcome (Tier 1). Instead, these patients underwent multiple reoperations, and an outcome better than Tier 3 could not be achieved.

High resistance rates were observed for cefuroxime and trimethoprim–sulfamethoxazol, and slightly lower resistance rates were observed for ceftriaxone, amoxicillin–clavulanic acid, ciprofloxacin, piperacillin–tazobactam and cefepime. In contrast, resistance to meropenem remained low, and no resistance was detected to gentamicin or levofloxacin. This finding is concerning, as antibiotic resistance, particularly in hospital-acquired infections, is a growing problem and could lead to prolonged hospital stays, increased healthcare costs and poor patient outcomes [14].

The treatment strategies in this study were diverse, with a predominance of two-stage revision procedures (65%) as the preferred method for managing septic revision cases. For GN and chronic PJIs, two-stage procedures are generally recommended [2,12,31]. We could not find a significant higher failure rate for chronic cases (*p* = 0.497). None of the initial applied treatment strategies showed a significantly worse outcome (*p* = 0.103). Due to the small sample size, no general recommendation for the right surgical approach can be made.

Two patients (11%) achieved successful outcomes (Tier 1), defined as the complete resolution of the infection and reimplantation. The majority (ten cases, 56%) were classified as Tier 3, indicating severe complications such as septic revision, amputation, resection arthroplasty or arthrodesis. Furthermore, six patients (33%) were classified as Tier 4 due to mortality. It should be mentioned that Tier 4 does not distinguish whether the death was causally related to the infection or not [24]. As most cases involve polymicrobial infections, it should be acknowledged that poor outcomes cannot be attributed solely to *Klebsiella*.

This suggests that infections involving *Klebsiella* may often be more complex and require a multi-faceted treatment approach. The polymicrobial nature of many *Klebsiella*-associated PJIs also supports the need for targeted antimicrobial therapies based on microbiological analysis, emphasizing the importance of obtaining accurate pre- and intraoperative cultures.

### Limitations

The limitations of this study are its small sample size and single-center nature, which restrict the external validity of the results. Moreover, the cohort comprises a selected population undergoing revision surgery, typically reflecting advanced disease stages, which further limits generalizability. The retrospective nature of this study causes potential biases related to data collection and treatment protocols. The long patient inclusion period represents an additional source of potential bias, as diagnostic approaches, surgical techniques, and antibiotic treatment strategies have continuously evolved over time. In most cases, *Klebsiella* was isolated only once; therefore, contamination cannot be entirely ruled out, despite careful sampling.

## 5. Conclusions

*Klebsiella*-associated PJIs accounted for 10.0% of Gram-negative and 1.0% of all culture-positive revision arthroplasties in our cohort. In total, 50% of the cases were chronic and 80% were polymicrobial, often involving additional Gram-positive or fungal pathogens. Two-stage revision was the predominant treatment strategy; however, only 11% achieved successful treatment (Tier 1). Antimicrobial resistance was frequent, as several isolates produced extended-spectrum beta-lactamases or were classified as multi-resistant Gram-negative bacteria. While penicillin and cephalosporins exhibited high resistance rates, meropenem demonstrated high susceptibility. Importantly, *Klebsiella* spp. was rarely identified as a primary infectious event and was predominantly detected in the context of complex or recurrent PJIs. These findings underline the clinical challenges of *Klebsiella*-associated PJIs and highlight the need for accurate microbiological diagnostics and optimized antimicrobial and surgical management.

## Figures and Tables

**Table 1:** Information about the cohort population, including demographic, infection, treatment, outcome and comorbidity characteristics.

	Culture Positive Revision-TJA (2008–2025) Cases *n*: 1925 Hip/Knee: 1125 (58%)/800 (42%)		
	Gram-Negative Positive Revision-TJA (2008–2025) Cases *n*: 201 (10.4% of 1925) Hip/Knee: 144/57 *Klebsiella*-Positive Revision-TJA 20 (1.0% of 1925) Hip/Knee: 11/9		
	Total	Hip	Knee
Male/Female	7/13	4/7	3/6
Age (Average/SD)	66.9/17.9	64.5/22.7	69.3/10.0
BMI (Average/SD)	29.4/4.9	28.9/5.4	29.9/4.5
ICM 2018			
Infected	11 (55%)	8	3
Inconclusive	7 (35%)	3	4
Not infected	2 (10%)	0	2
Characteristics			
UPIC	2 (10%)	0	2
Acute	8 (40%)	6	2
Chronic	10 (50%)	5	5
Monomicrobial	4 (20%)	1	3
Polymicrobial	16 (80%)	10	6
Treatment			
DAIR	4 (22%)	1	3
Single-Stage	1 (5%)	1	0
Two-Stage	13 (65%)	7	6
Tier classification			
I	2 (11%)	1	1
II	0 (0%)	0	0
III	10 (55%)	3	7
IV	6 (33%)	5	1
CCI (Average/SD)	4.4/2.4		
McPherson Score			
Infection Grade	I	II	III
	7	1	10
Systemic host grade	A	B	C
	3	12	5
Local extremity grade	I	II	III
	10	8	2

TJA: total joint arthroplasty; SD: standard deviation; UPIC: unexpected positive intraoperative culture; DAIR: debridement antibiotic and implant retention; CCI: Charlson Comorbidity Index; ICM: International Consensus Meeting.

**Table 2:** Information about the source and total amount of the collected and positive samples.

	Total		Hip		Knee	
	Total	Positive	Total	Positive	Total	Positive
Preoperative *Klebsiella* positive samples (*n*)	6		3		3	
**Perioperative tissue ** *total amount (average per surgery)*						
Tissue and Swabs	415 (3.4)	185 (1.8)	164 (2.9)	102 (2.1)	251 (3.7)	83 (1.6)
Sonication fluid	58 (0.5)	30 (0.3)	27 (0.5)	18 (0.3)	31 (0.6)	16 (0.3)
Samples in Monomicrobial Cases	39 (3.9)	13 (1.3)				
Samples in Polymicrobial Cases	434 (4.8)	172 (1.9)				

**Table 3:** Detailed description on the occurrence, frequency and antibiotic resistance profiles of all identified microorganisms. ESBL: extended-spectrum-beta lactamase; MRGN: multi-resistant Gram-negative.

	Total		Hip		Knee	
***Klebsiella* spp.**						
*Klebsiella pneumoniae*	18		11		7	
ESBL	8		4		4	
3 MRGN	4		2		2	
4 MRGN	1		1		0	
*Klebsiella oxytoca*	4		2		2	
3 MRGN	1		1		0	
**Co-Pathogens** (Polymicrobial Cases)						
**Gram-positive**	**32**		**17**		**15**	
*Staphylococcus epidermidis*	8		4		4	
*Enterococcus faecalis*	4		3		1	
*Cutibacterium acnes*	4		2		2	
*Corynebacterium*	4		2		2	
*Staphylococcus aureus [MRSA]*	3 [2]		1 [1]		2 [1]	
*Staphylococcus lugdunensis*	2		1		1	
*Staphylococcus hominis*	2		2		0	
*Staphylococcus haemolyticus*	1		1		0	
*Micrococcus luteus*	1		1		0	
*Staphylococcus caprae*	1		0		1	
*Staphylococcus haemolyticus*	1		0		1	
*Enterococcus faecium*	1		0		1	
**Gram-negative**	**13**		**6**		**7**	
*Citrobacter koseri*	3		2		1	
*Proteus mirabilis*	3		1		2	
*Pseud. aeruginosa*	3		1		2	
*Escherichia coli*	2		1		1	
*Morganella morganii*	1		1		0	
*Moraxella osloensis*	1		0		1	
**Fungi**	**4**		**3**		**1**	
*Candida albicans*	3		3		0	
*Candida parapsilosis*	1		0		1	

## Data Availability

The raw data supporting the conclusions of this article will be made available by the authors on request.

## Supplementary Materials

The following supporting information can be downloaded at https://www.mdpi.com/article/10.3390/pathogens15020164/s1. Table S1. Individual case characteristics of Klebsiella PJI., Strobe Guidelines.
