# Supplementary material for: The Role of Positive Klebsiella Culture in Revision Hip and Knee Arthroplasty
Source: Pathogens. 2026 Feb 3;15(2):164. doi: 10.3390/pathogens15020164 (PMC12943718; doi:10.3390/pathogens15020164)
Supplement: Supplementary file 1 [file pathogens-15-00164-s001.zip › pathogens-4089009-supplementary.pdf]

Supplementary Table S1. Individual case characteristics of *Klebsiella* associated PJI

Case	Joint	Klebsiella infection at:	Prior PJI	Time from primary implantation to Klebsiella infection in days	Klebsiella species	Diagnostic sample	Final surgical strategy	Antimicrobial regime	Duration in days	Outcome (Tier)
1	Hip	Re-Revision	Yes	1490	K. oxytoca	Intraoperative sample	Multiple-stage revision and Re-Implantation	Piperacillin/tazobactam → meropenem	71	4
2	Hip	Re-Revision	Yes	274	K. pneumoniae	Intraoperative sample	Multiple-stage revision and Re-Implantation	Ceftazidime/avibactam	7	NA
3	Hip	Primary Revision	No	16	K. pneumoniae	Intraoperative sample	Two-stage revision	Ceftriaxone, moxifloxacin, cephalexin, cotrimoxazole	115	1
4	Hip	Primary Revision	No	56	K. pneumoniae	Intraoperative sample	Multiple-stage revision and Re-Implantation	Meropenem	47	4
5	Hip	Re-Revision	Yes	791	K. pneumoniae	Intraoperative sample	Girdlestone procedure	Piperacillin/tazobactam, meropenem	56	3
6	Hip	Re-Revision	Yes	210	K. pneumoniae	Intraoperative sample	Girdlestone procedure	Meropenem, amoxicillin/clavulanic acid, cephalexin	83	4
7	Hip	Re-Revision	Yes	169	K. pneumoniae	Preoperative sample	Girdlestone procedure	Meropenem	23	4
8	Hip	Primary Revision	No	25	K. pneumoniae	Intraoperative sample	Wound revision with isolated mobile parts exchange	Meropenem	NA	3

9	Hip	Re-Revision	Yes	1179	K. pneumoniae	Preoperative sample	Girdlestone procedure	Ceftazidime/avibactam, aztreonam	NA	NA
10	Hip	Re-Revision	Yes	55	K. pneumoniae	Intraoperative sample	Girdlestone	Moxifloxacin	NA	4
11	Hip	Primary-Revision	No	NA	K. pneumoniae	Intraoperative sample	Multiple-stage revision and Re-Implantation	Piperacillin/tazobactam, meropenem	62	3
12	Knee	Primary-Revision	No	3771	K. pneumoniae	Intraoperative sample	Revision unicompartmental knee arthroplasty (UKA) to total knee arthroplasty (TKA)	Piperacillin/tazobactam	226	4
13	Knee	Re-Revision	Yes	4521	K. pneumoniae	Intraoperative sample	Wound revision with isolated mobile parts exchange	Ceftriaxone, ciprofloxacin	97	3
14	Knee	Re-Revision	Yes	5446	K. pneumoniae	Intraoperative sample	Knee arthrodesis	Meropenem	126	3
15	Knee	Re-Revision	Yes	4056	K. pneumoniae	Preoperative sample	Amputation	NA	NA	3
16	Knee	Re-Revision	Yes	4681	K. oxytoca	Preoperative sample	Knee arthrodesis	Meropenem	28	3
17	Knee	Primary Revision	No	800	K. pneumoniae	Preraoperative sample	One-stage revision	Moxifloxacin	27	1
18	Knee	Primary Revision	Yes	7531	K. pneumoniae	Intraoperative sample	Knee arthrodesis	Piperacillin/tazobactam, aztreonam, ceftazidime, ciprofloxacin	NA	3
19	Knee	Re-Revision	Yes	4454	K. pneumoniae	Preoperative sample	Amputation	Meropenem	90	3
20	Knee	Re-Revision	Yes	1559	K. pneumoniae	Intraoperative sample	Amputation	Meropenem	44	3

Tabelle 1: PJI periprosthetic joint infection, NA not available
